# Adding pharmacogenomics to the development of new marine-derived anticancer agents

**DOI:** 10.1186/1479-5876-4-3

**Published:** 2006-01-09

**Authors:** José Jimeno, Miguel Aracil, Juan Carlos Tercero

**Affiliations:** 1 PharmaMar R & D, Colmenar Viejo, Madrid, Spain

## Abstract

Nature has always been a highly productive tool in the development of anticancer therapies. Renewed interest in the potential of this tool has recently been sparked by the realization that the marine ecosystem can be used for the discovery and development of new compounds with clinical potential in advanced resistant tumors. These compounds can be incorporated into combination approaches in a chronic therapy scenario. Our marine anticancer program is using the sea to develop new agents with activity in resistant solid tumors and to identify new cellular targets for therapeutic intervention. This review describes the integration of different pharmacogenomic tools in the development of Yondelis™, Aplidin^® ^and Kahalalide F, three marine-derived compounds currently in Phase II or III development. Our results are reinforcing the targeted selectivity of these agents and opening the gates for customized therapies in cancer patients in the near future.

## Introduction

There is no doubt that major progress is being made in cancer treatment. The availability of non-cross-resistant regimens that are active in specific solid tumors means that several lines of therapy can be administered to patients. Thus, even in advanced stages, the disease can be chronified, and long-lasting survival can be achieved in selected responsive patients [1,2]. However, new standards of care are often the results of a trial-and-error approach, which means that large cohorts of patients are treated rather empirically in order to obtain clinical benefit in a relatively small proportion of patients.

The identification of specific molecular signatures and genetic polymorphisms that correlate with treatment outcome and treatment-associated toxicity has made it possible to propose "target" populations for cytotoxic therapy in patients with advanced solid tumors and hematologic malignancies [3,4]. The clinical impact of such an approach can be dramatic. For example, in the target population of lung adenocarcinoma patients harboring epidermal growth factor receptor (EGFR) tyrosine kinase domain mutations, treatment with EGFR tyrosine kinase inhibitors can achieve long-lasting responses in a high proportion of patients [5,6]. The incorporation of such molecular tools into contemporary drug development is mandatory if we are to optimize the clinical impact of new anticancer agents.

This review summarizes the information gathered from a translational research pharmacogenomic program that is being conducted with Yondelis™, Aplidin^® ^and Kahalalide F, three marine anticancer compounds that are active in pretreated cancer patients and are currently in phase II or III clinical development [7].

### YondelisTM (Trabectedin, ET-743)

**Figure 1:**
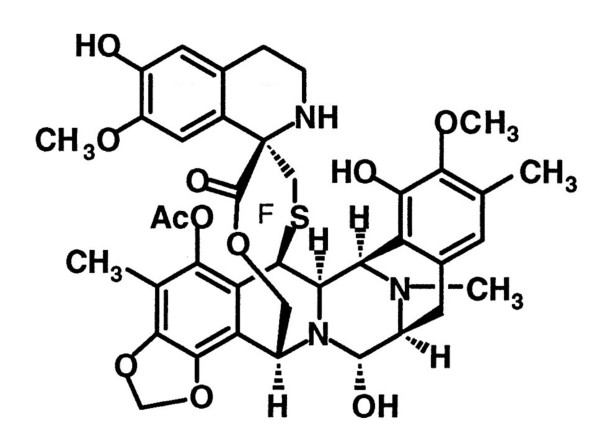
Chemical structure of Yondelis™.

Yondelis™ is a tetrahydroquinoline alkaloid identified in the Caribbean tunicate *Ecteinascidia turbinata *(Fig. 1). Earlier studies on the mechanism of action of Yondelis correlated its antitumor activity with a sequence selective binding to guanine in the minor groove of DNA and with a broad inhibition of activated transcription [8-11].

Yondelis™ is active in patients with advanced soft tissue sarcoma that is resistant or has relapsed after conventional therapies, with evidence of long-lasting objective responses and tumor control in 22% of cases [12-14]. No significant differences were observed in response to Yondelis™ between chemosensitive and chemoresistant patients. This finding, together with the large differences in survival time observed between responders and non-responders to Yondelis™, may indicate a differential molecular signature that correlates with clinical outcome to Yondelis™, at least in sarcoma patients. Such clinical evidence provided a rationale to look for a potential molecular predictive marker for response to Yondelis™ [15].

**Figure 2:**
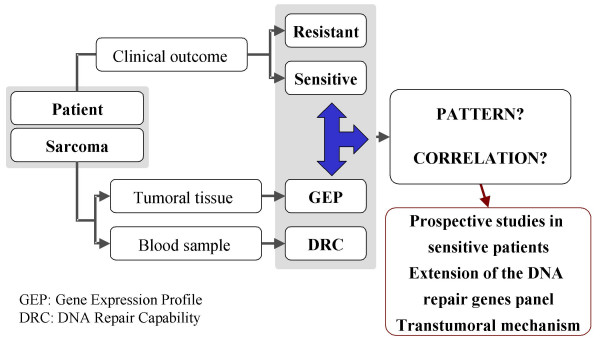
Strategy of the proposed pharmacogenomic program of Yondelis™ in sarcoma patients: Gene expression profiles of tumor samples from sarcoma patients treated with Yondelis will be retrospectively analyzed and correlated with their clinical outcome. The DNA repair capability will be analyzed in blood samples of the same patients in order to be used as surrogate marker of response. The putative correlation found between GEP and clinical outcome will be prospectively analyzed in sarcoma patients and further studied in other tumors.

In addition to the clinical evidence, strong evidence from the pre-clinical studies indicates that an efficient transcription coupled (TC) nucleotide excision repair (NER) system is crucial to the antitumor activity of Yondelis™ [16,17]. TC-NER involves genes that are deficient in rare inborn disorders such as Cockayne syndrome and xeroderma pigmentosum. Patients with mutations in the TC-NER system exhibit an increased predisposition to develop cancer [18]. The TC-NER system is involved in the repair of bulky adducts induced by classical alkylating agents and by platin salts. In fact, cytotoxic drugs are less active in TC-NER-efficient tumors, in contrast to Yondelis™, which requires an efficient TC-NER system. This differential pharmacodynamic effect led us to investigate a possible correlation between the mRNA expression levels of specific DNA repair genes and the clinical outcome of patients treated with Yondelis™. As a result, a pharmacogenomic model was implemented to identify a customized therapy for prospective validation (Fig. 2).

The first model included the molecular characterization of a panel of low passage human sarcoma cell lines explanted from chemonaive patients [19], which included different sarcoma sub-types; in this panel of sarcoma cell lines, an IC_50 _> 1 nM was established as the cut-off for resistance. This concentration may be achieved and maintained for more than 72 hours in plasma of sarcoma patients treated below the recommended clinical dose incorporated in the phase II trials, and is therefore considered to be therapeutically appropriate for this experimental study [20]. The *in vitro *results indicated a pattern of primary sensitivity and resistance in cell lines that was in line with the fact that sarcoma patients can be either highly sensitive or fully resistant to Yondelis™. Furthermore, a complete lack of cross-resistance with doxorubicin, the drug used as first line treatment in sarcoma patients, was observed. In addition, although mutated p53 is associated with resistance to conventional anticancer therapies and is considered to be a poor prognostic factor for outcome in cancer patients [21], studies suggest a direct correlation between mutated p53 and *in vitro *sensitivity to Yondelis™. These interesting early results are currently being validated in a larger human sarcoma panel.

The full panel of human sarcoma cell lines was further exposed to the clinically relevant concentration of 10 nM Yondelis™ during different incubation times. RNA from the cells was extracted at 0, 6, 24, 48 and 72 hours, and baseline and dynamic gene expression profiles (GEP) were assessed [22], using the Oncochip cDNA microarray (developed by the Spanish National Center for Cancer Research), which includes clones of 6388 cancer related genes [23]. GEP revealed upregulation of 86 genes and downregulation of 244 genes in the cell line panel after *in vitro *exposure to pharmacological concentrations of Yondelis™ for 24 hours. This gene expression signature induced after Yondelis™ treatment identified a group of genes involved in cell cycle control, DNA damage response and apoptosis.

Based on the differential baseline and dynamic GEPs observed in sensitive and resistant sarcoma cell lines, we have drawn up a set of potential genetic markers of response to Yondelis™ and are currently using quantitative RT-PCR to assess their mRNA expression levels and protein expression in tissue arrays from sarcoma patients [24] (Table 1).

**Table 1:** Candidate markers of response* to Yondelis

**Gene ID**	**Description**
IFITM2	interfer on induced transmembrane protein 2 (1-8D)
TP53	tum or protein p53 (Li-Fraumeni syndrome)
COL5A2	collagen, type V, alpha 2
JUNB	jun B proto-oncogene
BST2	bone marrow stromal cell antigen 2
HHEX	hematopoietically expressed homeobox
SERPINA3	serine (or cysteine) proteinase inhibitor, clade A (alpha-1 antiproteinase, antitrypsin), member 3
ATF3	activating transcription factor 3
ABL1	v-abl Abelson murine leukemia viral oncogene homolog 1
BRCA1	breast cancer 1, early onset
ERCC2 XPD	excision repair cross-complementing rodent repair deficiency, complementation group 2 (xeroderma pigmentosum D)
ERCC3 XPB	excision repair cross-complementing rodent repair deficiency, complementation group 3 (xeroderma pigmentosum group B complementing)
PCNA	proliferating cell nuclear antigen
POLD3	polymerase (DNA directed), delta 3 (Interim)
POLR2G	polymerase (RNA) II (DNA directed) polypeptide G
PRKDC	protein kinase, DNA-activated, catalytic polypeptide
PTTG1	pituitary tumor-transforming 1
RAD17	RAD17 homolog (S. pombe)

* Genes were selected based on several *in vitro *experiments of differential baseline (resistant cells vs sensitive cells) and dynamic (treated cells vs untreated cells) Gene Expression Profiles observed in sarcoma cell lines. Their mRNA and protein expression levels are currently being analyzed in tumor samples from sarcoma patients and correlated with clinical outcome in order to validate them as potential markers of response to Yondelis.

The correlation between DNA repair capacity and sensitivity to Yondelis™ in experimental models led us to conduct a retrospective study in tumor samples from advanced resistant or relapsed sarcoma patients treated with Yondelis™ to correlate DNA repair capacity with treatment outcome [25]. RNA was extracted from paraffin-embedded tumors to determine the mRNA expression levels of ERCC1, BRCA1 and XPD by quantitative RT-PCR. ERRC1 (subunit of a 5'-endonuclease) and XPD (subunit of a DNA helicase) are involved in the NER pathway, and upregulation of these genes in tumors results in a poor outcome to treatment with DNA-interacting agents [26]. BRCA1 is involved in homologous recombination repair and non-homologous end joining in response to double-stranded breaks DNA damage [27]. In lung cancer, upregulation of BRCA1 has been linked to both increased resistance to cisplatin and increased sensitivity to taxanes [28,29]. Our retrospective study included 45 heavily pretreated sarcoma patients; the overall objective response rate was 11%, progression free survival at 6 months (PFS6) was attained by 22% of patients, and the median overall survival was 11.8 months. These findings are fully consistent with earlier results [15]. The mRNA expression studies indicate that low (below the median) levels of BRCA1 correlate with a higher objective response rate (24% in low levels vs 6% in high levels). Moreover, 36% of patients with low levels attained PFS6, compared to only 6% of those with high levels (P = 0.06). Median survival was 16.8 months in patients with low levels and 6 months in those with high levels (P = 0.02). In contrast, patients with high mRNA expression levels of XPD and ERCC1 showed a tendency towards better response rate and superior PFS6 figures.

In summary, our translational pharmacogenomic research with Yondelis™ in sarcoma has identified a set of genes that correlate with the *in vitro *response to pharmacological concentrations of the compound. The data gathered in our retrospective analysis of tumor samples from sarcoma patients demonstrate that clinical response to Yondelis™ is modulated by the molecular profile of several DNA repair genes in a unique differential pattern not applicable to other DNA-interacting agents, such as doxorubicin, a drug often used in the treatment of sarcomas.

### Aplidin^® ^(Plitidepsin, APLD)

**Figure 3:**
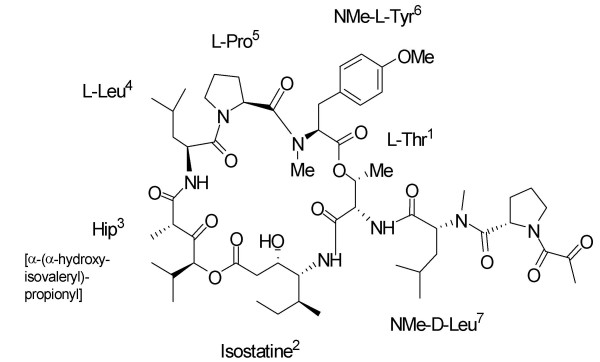
Chemical structure of Aplidin^®^.

Aplidin^® ^(Fig 3) is a marine depsipeptide found in the Mediterranean tunicate *Aplidium albicans*. Aplidin^® ^is an NCI COMPARE compound that induces G1 cell-cycle arrest and very rapid p53- and MDR-independent apoptosis [30]. Aplidin^®^-induced apoptosis seems to be mediated through mitochondrial pathways involving JNK-dependent oxidative stress, activation of p38 and protein C delta kinases, and glutathion depletion [31,32]. Interestingly, Aplidin^® ^affects the endothelial vascular growth factor (VEGF) pathway, which may account for its apoptotic activity. Aplidin^® ^affects VEGF secretion by inducing a two-fold inhibition of the autocrine loop and a downregulation of the VEGF flt-1 receptor in acute lymphoblastic leukemia cell lines [33]. However, the reported accumulation of the VEGF mRNA transcript suggests a selective impact on VEGF secretion but not VEGF production [34]. An *in vivo *study in anaplastic thyroid carcinoma studied the changes in GEP in response to Aplidin^® ^and found a potential effect on the expression of angiogenesis-related genes. RNA was extracted from control and Aplidin^®^-treated tumors and hybridized on a cDNA microarray (GEarray Q series Human Angiogenesis Array (HS-009) containing probes from 96 genes involved in angiogenesis. The results showed a dramatic reduction of VEGF-D expression in the tumors of treated animals as well as a complete loss of other angiogenesis-related genes such as hypoxia inducible factor-1, vasostatin and cadherin. In contrast, *in vivo *exposure to Aplidin^® ^led to an increase in the levels of proteins involved in apoptosis, such as PARP-85 and caspase 8 [35].

Aplidin^® ^has demonstrated activity and feasibility in chemoresistant adult cancer patients, with a remarkably low rate of bone marrow and hematological toxicity. It is currently in active phase II development in solid tumors and hematological malignancies.

**Figure 4:**
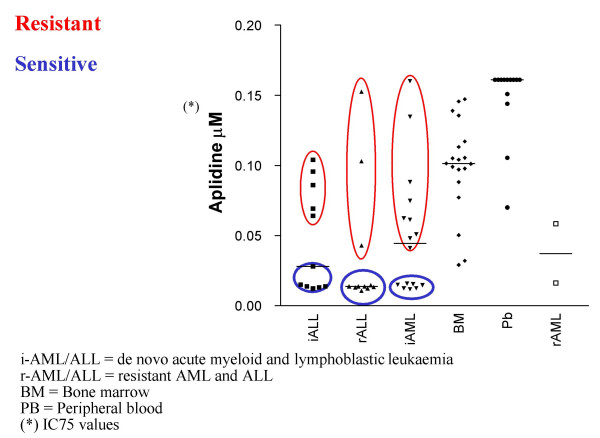
Aplidin^® ^*in vitro *differentiation of sensitive and resistant leukemic blasts.

The pharmacodynamic impact of Aplidin^® ^in the VEGF loop, as well as its low rate of hematotoxicity, led us to investigate its effect in hematological malignancies, especially in acute leukemia, lymphomas and multiple myeloma. A series of experimental studies conducted in leukemia blasts from pediatric and adult leukemia patients confirmed that Aplidin^® ^is able to induce massive apoptosis at therapeutic concentrations of 5–10 nM [36], with no full cross-resistance to conventional anticancer agents except gemcitabine [37]. The reason for this crossresistance is so far unexplained and further experiments are planned to elucidate this finding. The *in vitro *data demonstrates clear differences between blasts from leukemia patients who are highly sensitive or resistant to Aplidin^® ^(Fig. 4).

**Figure 5:**
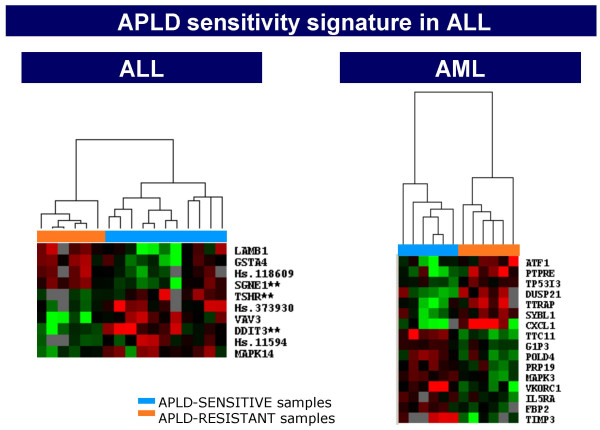
Molecular signatures of sensitivity to Aplidin (APLD) in acute lymphoblastic leukemia (ALL) and acute myeloid leukemia (AML) patient blasts.

These results with leukemic blasts enabled us to develop a pharmacogenomic model of a molecular fingerprint for sensitivity or resistance of leukemic blasts to Aplidin^® ^. RNA was isolated from patient blasts and specific GEPs were analyzed in the cDNA cancer specific array Oncochip^®^. The results [38,39] indicated a GEP signature specific for sensitive and resistant acute myeloid and acute lymphoblastic leukemic blasts (Fig. 5). This model is being incorporated into the ongoing phase II trial with Aplidin^® ^in resistant and relapsed acute lymphoblastic leukemia. If a correlation is observed between baseline GEP and patient outcome, a target population can be identified for future developmental strategies. The same approach has been implemented in the phase II trial in pretreated multiple myeloma.

Studies of Aplidin^® ^in combination with other agents in hematological malignances are currently ongoing. In addition, GEP assessment is being incorporated in *in vivo *studies to investigate the molecular basis behind the strong synergism of Aplidin^® ^and ARA-C in leukemia and lymphoma models [40]. Initial GEP analyses in these experimental models, using the U133A GeneChip (Affymetrix), suggest that Aplidin^®^-ARA-C combination affects multiple pathways including MAP kinase pathway, WNT signaling pathway, cell cycle regulators and the tricarboxylic acid cycle. Interestingly, the GEPs from the *in vivo *combination APLD-ARA-C studies demonstrate a significant downregulation of cytidine deaminase, an enzyme that deactivates ARA-C.

### Kahalalide F

**Figure 6:**
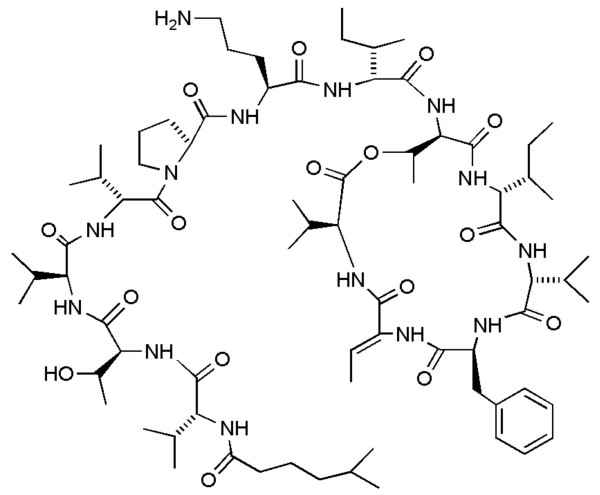
Chemical structure of Kahalalide F.

Kahalalide-F is a cyclic peptide found in the Hawaiian nudibranch *E. rufescens *(Fig. 6). Kahalalide-F is an NCI-COMPARE compound that induces sub G1 cell-cycle arrest and cytotoxicity independently of MDR, Her2, p53 and blc-2 [41]. It has completed the phase I program with evidence of a positive therapeutic index in patients with solid tumors and prostate cancer [7,42] and is under active phase II development in liver cancer, melanoma and non-small cell lung cancer.

The COMPARE analysis in a panel of 60 human cancer cell lines genetically and molecularly characterized for cell proliferation pathways has included Kahalalide-F in the list of new chemical entities that interact with the Erb/Her-neu pathway [43]. This specific interaction has been described in a translational program that has confirmed a selective downregulation of ErbB3 expression by KF treatment. Expression of ErbB3 is seen in many of the same tumor types that overexpress ErbB2, including breast, bladder and melanomas. The involvement of ErbB3 overexpression in the genesis and maintenance of lung adenocarcinomas has been recently suggested [44]. ErbB3 is a major recruiter of phosphoinositide 3-kinase (PI3K); it often couples to other ErbB receptors to activate the downstream Akt pathway and promotes the cancer phenotype. Sensitivity to Kahalalide-F significantly correlated with baseline expression levels of ErbB3 (HER3), but not of other ErbB receptors, in a panel of established cell lines from different origins. Furthermore, the downstream PI3K/Akt pathway coupled to ErbB3 receptor is also affected by KF treatment. KF decreases phosphorylated Akt levels and this reduction is associated with cytotoxicity in Kahalalide-F-sensitive cell lines [45]. These findings suggest that ErbB3 may be a potential marker for Kahalalide-F sensitivity in patients.

## Discussion

In this review, we have described the different approaches that have led us to propose pharmacogenomic models for three innovative marine anticancer compounds that are under clinical development.

The dynamic GEP study with Yondelis™ in human sarcoma cell lines has confirmed its impact on transcriptional machinery, shedding light on the molecular basis for its antiproliferative effects. The *in vitro *model has also been instrumental in the identification of differential baseline gene signatures that correlate with sensitivity or resistance to Yondelis™, thus reducing the set of genes that can be considered potential biomarkers for response validation studies. Tumor samples from Yondelis™-treated sarcoma patients are being collected to build tissue arrays to confirm the impact of gene expression levels on patient response. Preliminary data from a small cohort of heavily pretreated sarcoma patients indicates that the SNPs and the mRNA expression levels of a number of DNA repair genes induce a differential response to Yondelis™. These interesting findings confirm that sensitivity to Yondelis™ bears a unique correlation with the NER and the homologous recombination repair systems. Such findings are in contrast with the data on platin salts and other DNA binding agents [26,28] and on taxanes [27], providing support for the use of Yondelis™ in combination strategies [46-48] to increase disease control in highly resistant patients. These clinically relevant findings are now being validated in a second retrospective cohort of more than 100 sarcoma patients treated with Yondelis™ and will be further confirmed in prospective clinical validation studies.

The impact of DNA repair capacity on treatment outcome with Yondelis™ is not limited to sarcoma but is applicable to other tumors as well. For this reason, pharmacogenomic studies in other tumors that are highly sensitive to Yondelis™, such as relapsed ovarian cancer [49], are strongly encouraged.

Pharmacogenomic studies examining baseline GEPs that may be linked to the *in vitro *response of leukemic blasts to Aplidin^® ^have been incorporated into phase II studies in relapsed lymphoblastic leukemia and multiple myeloma. Retrospective studies to identify differential GEPs in other solid tumors that are sensitive to Aplidin^®^, such as advanced pretreated melanoma [50], are also ongoing. "Preclinical pharmacogenomics" have been instrumental to understand the molecular basis behind Aplidin^® ^apoptotic effects as well as to provide a basis for combination studies. In fact, recent *in vivo *dynamic GEP studies have confirmed a major impact on angiogenesis-related genes, thus providing a rationale for combining Aplidin^® ^with other VEGF-interacting agents.

Experimental evidence for a selective impact of Kahalalide-F on the ErbB3 pathway has provided a basis for studies to validate these findings in extremely sensitive or resistant *in vitro *and *in vivo *models. Results from these studies could justify further explorations in tumor samples from patients. This experimental data has established a basis for *in vitro *and *in vivo *studies combining Kahalalide F with ErbB kinase inhibitors and ErbB monoclonal antibodies.

## Conclusion

Our journey into the new era of pharmacogenomics has confirmed that these new tools can potentially elucidate the molecular basis behind individual differences in the pharmacodynamic effects of marine anticancer compounds and enable us to design customized models for therapeutic intervention in cancer patients. A better understanding of the molecular determinants of therapeutic response will help identify patients at risk for severe toxicities or those more likely to respond to a given therapeutic regimen [51], thus paving the way for customized anticancer therapy to become a reality in the near future. A proactive interaction between researchers, the pharmaceutical sector and government regulating agencies is crucial to the incorporation of this challenging new tool in clinical medicine.
